# Methyl 4-(5-meth­oxy-1*H*-indol-3-yl)benzoate

**DOI:** 10.1107/S1600536811052135

**Published:** 2011-12-17

**Authors:** Cui-Ping Wang, Jiang-Long Yu, Zhi-Qiang Zhang, Jing-Bo Yan

**Affiliations:** a School of Chemical Engineering, University of Science and Technology Liaoning, Anshan 114051, People’s Republic of China

## Abstract

In the title compound, C_17_H_15_NO_3_, the dihedral angle between the benzene ring and the indole ring system is 22.5 (3)°. In the crystal, mol­ecules are linked by N—H⋯π and C—H⋯O inter­actions.

## Related literature

For background to the catalysed aryl­ation of indoles, see: Zhang *et al.* (2007 ▶). For reference bond-length data, see: Allen *et al.* (1987 ▶).
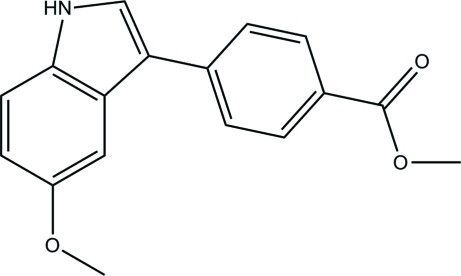


## Experimental

### 

#### Crystal data

C_17_H_15_NO_3_
*M* *_r_* = 281.30
Monoclinic, 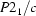

*a* = 15.023 (8) Å
*b* = 5.871 (3) Å
*c* = 16.867 (9) Å
β = 113.721 (6)°
*V* = 1361.9 (12) Å^3^
*Z* = 4
Mo *K*α radiation
μ = 0.10 mm^−1^
*T* = 113 K
0.20 × 0.16 × 0.14 mm

#### Data collection

Rigaku Saturn724 CCD diffractometer
Absorption correction: multi-scan (*CrystalClear*; Rigaku, 2007 ▶) *T*_min_ = 0.981, *T*_max_ = 0.987
13161 measured reflections
3241 independent reflections
2460 reflections with *I* > 2σ(*I*)
*R*_int_ = 0.038

#### Refinement

*R*[*F* ^2^ > 2σ(*F* ^2^)] = 0.037
*wR*(*F* ^2^) = 0.110
*S* = 1.06
3241 reflections
196 parameters
1 restraint
H atoms treated by a mixture of independent and constrained refinement
Δρ_max_ = 0.24 e Å^−3^
Δρ_min_ = −0.25 e Å^−3^

### 

Data collection: *CrystalClear* (Rigaku, 2007 ▶); cell refinement: *CrystalClear*; data reduction: *CrystalClear*; program(s) used to solve structure: *SHELXS97* (Sheldrick, 2008 ▶); program(s) used to refine structure: *SHELXL97* (Sheldrick, 2008 ▶); molecular graphics: *SHELXTL* (Sheldrick, 2008 ▶); software used to prepare material for publication: *SHELXTL*.

## Supplementary Material

Crystal structure: contains datablock(s) global, I. DOI: 10.1107/S1600536811052135/hb6526sup1.cif

Structure factors: contains datablock(s) I. DOI: 10.1107/S1600536811052135/hb6526Isup2.hkl

Supplementary material file. DOI: 10.1107/S1600536811052135/hb6526Isup3.cml

Additional supplementary materials:  crystallographic information; 3D view; checkCIF report

## Figures and Tables

**Table 1:** Hydrogen-bond geometry (Å, °) *Cg*2 is the centroid ofthe C2–C5/C8/C9 ring.

*D*—H⋯*A*	*D*—H	H⋯*A*	*D*⋯*A*	*D*—H⋯*A*
N1—H1⋯*Cg*2^i^	0.90 (1)	2.54 (2)	3.295 (2)	142 (1)
C6—H6⋯O1^ii^	0.95	2.43	3.369 (2)	172
C17—H17*B*⋯O2^iii^	0.98	2.60	3.484 (2)	150

Symmetry codes: (i) 
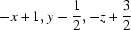
; (ii) 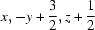; (iii) 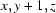.

**Fig. 1.**
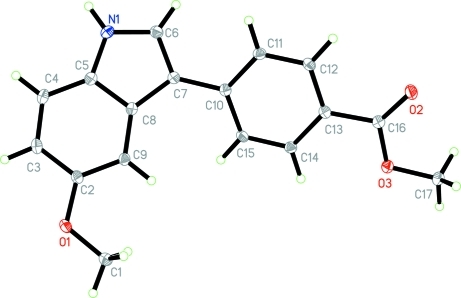
The molecular structure of the title compound, showing 30% probability displacement ellipsoids for the non-hydrogen atoms.

## supplementary crystallographic information

### Comment

In 2007, our group reported direct palladium-catalyzed C-3 arylation of indoles (Zhang *et al.*, 2007). As an extension of this work, we now report the synthesis and crystal structure of the title compound, (I), (Fig. 1).

The dihedral angle between the benzene ring and the indole ring is 22.5 (3)°. All the bond values are within normal ranges (Allen *et al.*, 1987).    In the crystal, N—H···π and C—H···O interactions occur (Table 1).

### Experimental

A mixture of 5-methoxy-1*H*-indole (0.5 mmol), 1-(4-bromophenyl)ethanone (0.6 mmol), potassium carbonate (1.5 mmol) and (^t^Bu)_2_P(OH)]_2_PdCl_2_ (abbreviated as POPd, 0.025 mmol) was stirred and refluxed in 2 ml of dioxane under nitrogen atmosphere for 24 h. The reaction mixture was allowed to cool to room temperature, quenched with water and extracted with EtOAc. The combined organic layers were washed with brine and dried over MgSO_4_, and the solvent was removed under vacuum. The residue was purified by chromatography on silica gel eluting with hexane/EtOAc (5/1 by vol.) to give light yellow power in 54.0%, m.p.123.0–124.8 °C. Colourless prisms of (I) were grown by slow evaporation of a solution in chloroform/ethanol (1:1).

### Refinement

Atom H1 was located in a difference Fourier map and refined isotropically, with the N—H distance restrained to 0.90 (1) Å. The remaining H atoms were placed in calculated positions (C—H = 0.95–0.98 Å) and refined as riding with *U*_iso_(H) = 1.2*U*_eq_(C) and 1.5 *U*_eq_(C1 and C17).

### Crystal data

**Table:**

C_17_H_15_NO_3_	*F*(000) = 592
*M**_r_* = 281.30	*D*_x_ = 1.372 Mg m^−^^3^
Monoclinic, *P*2_1_/*c*	Mo *K*α radiation, λ = 0.71073 Å
*a* = 15.023 (8) Å	Cell parameters from 4649 reflections
*b* = 5.871 (3) Å	θ = 1.5–27.9°
*c* = 16.867 (9) Å	µ = 0.10 mm^−^^1^
β = 113.721 (6)°	*T* = 113 K
*V* = 1361.9 (12) Å^3^	Prism, colorless
*Z* = 4	0.20 × 0.16 × 0.14 mm

### Data collection

**Table:**

Rigaku Saturn724 CCD diffractometer	3241 independent reflections
Radiation source: rotating anode	2460 reflections with *I* > 2σ(*I*)
multilayer	*R*_int_ = 0.038
Detector resolution: 14.22 pixels mm^-1^	θ_max_ = 27.9°, θ_min_ = 1.5°
ω and φ scans	*h* = −19→19
Absorption correction: multi-scan (*CrystalClear*; Rigaku, 2007)	*k* = −7→7
*T*_min_ = 0.981, *T*_max_ = 0.987	*l* = −22→22
13161 measured reflections	

### Refinement

**Table:**

Refinement on *F*^2^	Primary atom site location: structure-invariant direct methods
Least-squares matrix: full	Secondary atom site location: difference Fourier map
*R*[*F*^2^ > 2σ(*F*^2^)] = 0.037	Hydrogen site location: inferred from neighbouring sites
*wR*(*F*^2^) = 0.110	H atoms treated by a mixture of independent and constrained refinement
*S* = 1.06	*w* = 1/[σ^2^(*F*_o_^2^) + (0.0702*P*)^2^] where *P* = (*F*_o_^2^ + 2*F*_c_^2^)/3
3241 reflections	(Δ/σ)_max_ = 0.001
196 parameters	Δρ_max_ = 0.24 e Å^−^^3^
1 restraint	Δρ_min_ = −0.25 e Å^−^^3^

### Special details

**Table:**

Geometry. All e.s.d.'s (except the e.s.d. in the dihedral angle between two l.s. planes) are estimated using the full covariance matrix. The cell e.s.d.'s are taken into account individually in the estimation of e.s.d.'s in distances, angles and torsion angles; correlations between e.s.d.'s in cell parameters are only used when they are defined by crystal symmetry. An approximate (isotropic) treatment of cell e.s.d.'s is used for estimating e.s.d.'s involving l.s. planes.
Refinement. Refinement of *F*^2^ against ALL reflections. The weighted *R*-factor *wR* and goodness of fit *S* are based on *F*^2^, conventional *R*-factors *R* are based on *F*, with *F* set to zero for negative *F*^2^. The threshold expression of *F*^2^ > σ(*F*^2^) is used only for calculating *R*-factors(gt) *etc*. and is not relevant to the choice of reflections for refinement. *R*-factors based on *F*^2^ are statistically about twice as large as those based on *F*, and *R*- factors based on ALL data will be even larger.

### Fractional atomic coordinates and isotropic or equivalent isotropic displacement parameters (Å^2^)

**Table:**

	*x*	*y*	*z*	*U*_iso_*/*U*_eq_	
O1	0.65330 (5)	1.06706 (14)	0.57107 (5)	0.0256 (2)	
O2	1.06010 (5)	1.07061 (13)	1.21305 (5)	0.0268 (2)	
O3	1.03480 (5)	1.38309 (12)	1.12988 (5)	0.0231 (2)	
N1	0.57543 (7)	0.50593 (17)	0.79506 (6)	0.0252 (2)	
C1	0.72517 (8)	1.2391 (2)	0.60676 (7)	0.0266 (3)	
H1A	0.7069	1.3383	0.6445	0.040*	
H1B	0.7300	1.3297	0.5598	0.040*	
H1C	0.7881	1.1678	0.6405	0.040*	
C2	0.63635 (7)	0.92664 (19)	0.62868 (7)	0.0201 (2)	
C3	0.56197 (8)	0.76524 (19)	0.59007 (7)	0.0233 (3)	
H3	0.5285	0.7610	0.5288	0.028*	
C4	0.53738 (7)	0.61387 (19)	0.64008 (7)	0.0232 (3)	
H4	0.4883	0.5024	0.6144	0.028*	
C5	0.58711 (7)	0.62954 (18)	0.73024 (7)	0.0206 (2)	
C6	0.63929 (8)	0.58338 (19)	0.87366 (7)	0.0230 (2)	
H6	0.6447	0.5255	0.9280	0.028*	
C7	0.69470 (7)	0.75701 (17)	0.86292 (7)	0.0188 (2)	
C8	0.66109 (7)	0.79141 (17)	0.77034 (7)	0.0177 (2)	
C9	0.68678 (7)	0.94025 (18)	0.71752 (6)	0.0191 (2)	
H9	0.7377	1.0478	0.7424	0.023*	
C10	0.77498 (7)	0.86947 (18)	0.93408 (7)	0.0182 (2)	
C11	0.82087 (7)	0.75877 (18)	1.01399 (7)	0.0204 (2)	
H11	0.7983	0.6129	1.0219	0.024*	
C12	0.89807 (7)	0.85678 (18)	1.08147 (7)	0.0202 (2)	
H12	0.9277	0.7784	1.1350	0.024*	
C13	0.93258 (7)	1.06998 (18)	1.07123 (7)	0.0186 (2)	
C14	0.88750 (7)	1.18392 (18)	0.99234 (7)	0.0197 (2)	
H14	0.9106	1.3292	0.9845	0.024*	
C15	0.80911 (7)	1.08586 (18)	0.92535 (7)	0.0199 (2)	
H15	0.7780	1.1670	0.8726	0.024*	
C16	1.01559 (7)	1.16901 (18)	1.14560 (7)	0.0194 (2)	
C17	1.11194 (8)	1.4968 (2)	1.20010 (7)	0.0237 (3)	
H17A	1.1040	1.4704	1.2543	0.036*	
H17B	1.1092	1.6607	1.1883	0.036*	
H17C	1.1749	1.4365	1.2053	0.036*	
H1	0.5318 (9)	0.3932 (19)	0.7867 (10)	0.047 (4)*	

### Atomic displacement parameters (Å^2^)

**Table:**

	*U*^11^	*U*^22^	*U*^33^	*U*^12^	*U*^13^	*U*^23^
O1	0.0261 (4)	0.0340 (5)	0.0151 (4)	−0.0048 (3)	0.0069 (3)	0.0006 (3)
O2	0.0269 (4)	0.0248 (4)	0.0210 (4)	0.0003 (3)	0.0017 (3)	0.0020 (3)
O3	0.0247 (4)	0.0209 (4)	0.0202 (4)	−0.0035 (3)	0.0056 (3)	−0.0013 (3)
N1	0.0233 (5)	0.0248 (5)	0.0264 (5)	−0.0070 (4)	0.0090 (4)	−0.0006 (4)
C1	0.0273 (6)	0.0322 (7)	0.0208 (6)	−0.0051 (5)	0.0102 (5)	0.0010 (5)
C2	0.0189 (5)	0.0235 (6)	0.0182 (5)	0.0021 (4)	0.0076 (4)	0.0000 (4)
C3	0.0198 (5)	0.0285 (6)	0.0181 (5)	0.0015 (4)	0.0041 (4)	−0.0046 (4)
C4	0.0188 (5)	0.0235 (6)	0.0247 (6)	−0.0020 (4)	0.0058 (4)	−0.0060 (4)
C5	0.0181 (5)	0.0201 (5)	0.0231 (6)	0.0004 (4)	0.0078 (4)	−0.0013 (4)
C6	0.0227 (5)	0.0248 (6)	0.0215 (5)	−0.0009 (4)	0.0090 (4)	0.0018 (4)
C7	0.0188 (5)	0.0190 (5)	0.0190 (5)	0.0011 (4)	0.0081 (4)	−0.0002 (4)
C8	0.0147 (5)	0.0187 (5)	0.0190 (5)	0.0019 (4)	0.0062 (4)	−0.0012 (4)
C9	0.0162 (5)	0.0220 (5)	0.0176 (5)	−0.0008 (4)	0.0052 (4)	−0.0012 (4)
C10	0.0181 (5)	0.0205 (5)	0.0180 (5)	0.0015 (4)	0.0092 (4)	−0.0014 (4)
C11	0.0227 (5)	0.0174 (5)	0.0216 (5)	−0.0001 (4)	0.0095 (4)	0.0007 (4)
C12	0.0222 (5)	0.0201 (6)	0.0179 (5)	0.0041 (4)	0.0075 (4)	0.0023 (4)
C13	0.0194 (5)	0.0202 (6)	0.0175 (5)	0.0025 (4)	0.0087 (4)	−0.0013 (4)
C14	0.0233 (5)	0.0188 (5)	0.0188 (5)	−0.0007 (4)	0.0102 (4)	−0.0006 (4)
C15	0.0239 (5)	0.0208 (6)	0.0158 (5)	0.0010 (4)	0.0089 (4)	0.0015 (4)
C16	0.0196 (5)	0.0198 (5)	0.0204 (5)	0.0018 (4)	0.0097 (4)	−0.0011 (4)
C17	0.0234 (5)	0.0235 (6)	0.0221 (6)	−0.0035 (4)	0.0069 (4)	−0.0031 (4)

### Geometric parameters (Å, °)

**Table:**

O1—C2	1.3739 (14)	C6—H6	0.9500
O1—C1	1.4232 (14)	C7—C8	1.4491 (16)
O2—C16	1.2095 (13)	C7—C10	1.4718 (15)
O3—C16	1.3397 (14)	C8—C9	1.4082 (15)
O3—C17	1.4449 (13)	C9—H9	0.9500
N1—C6	1.3643 (15)	C10—C15	1.3997 (16)
N1—C5	1.3811 (15)	C10—C11	1.4025 (15)
N1—H1	0.902 (8)	C11—C12	1.3818 (15)
C1—H1A	0.9800	C11—H11	0.9500
C1—H1B	0.9800	C12—C13	1.3919 (16)
C1—H1C	0.9800	C12—H12	0.9500
C2—C9	1.3828 (15)	C13—C14	1.3970 (15)
C2—C3	1.4089 (16)	C13—C16	1.4859 (15)
C3—C4	1.3742 (16)	C14—C15	1.3872 (15)
C3—H3	0.9500	C14—H14	0.9500
C4—C5	1.4020 (17)	C15—H15	0.9500
C4—H4	0.9500	C17—H17A	0.9800
C5—C8	1.4110 (15)	C17—H17B	0.9800
C6—C7	1.3733 (15)	C17—H17C	0.9800
			
C2—O1—C1	116.85 (9)	C5—C8—C7	106.77 (9)
C16—O3—C17	115.91 (9)	C2—C9—C8	118.67 (10)
C6—N1—C5	109.30 (10)	C2—C9—H9	120.7
C6—N1—H1	125.4 (10)	C8—C9—H9	120.7
C5—N1—H1	125.3 (10)	C15—C10—C11	117.54 (10)
O1—C1—H1A	109.5	C15—C10—C7	122.41 (10)
O1—C1—H1B	109.5	C11—C10—C7	120.04 (10)
H1A—C1—H1B	109.5	C12—C11—C10	121.59 (10)
O1—C1—H1C	109.5	C12—C11—H11	119.2
H1A—C1—H1C	109.5	C10—C11—H11	119.2
H1B—C1—H1C	109.5	C11—C12—C13	120.22 (10)
O1—C2—C9	123.72 (10)	C11—C12—H12	119.9
O1—C2—C3	114.50 (10)	C13—C12—H12	119.9
C9—C2—C3	121.78 (10)	C12—C13—C14	119.12 (10)
C4—C3—C2	120.68 (10)	C12—C13—C16	118.36 (10)
C4—C3—H3	119.7	C14—C13—C16	122.51 (10)
C2—C3—H3	119.7	C15—C14—C13	120.30 (10)
C3—C4—C5	117.76 (10)	C15—C14—H14	119.8
C3—C4—H4	121.1	C13—C14—H14	119.8
C5—C4—H4	121.1	C14—C15—C10	121.18 (10)
N1—C5—C4	129.97 (11)	C14—C15—H15	119.4
N1—C5—C8	107.51 (10)	C10—C15—H15	119.4
C4—C5—C8	122.51 (10)	O2—C16—O3	123.55 (10)
N1—C6—C7	110.23 (10)	O2—C16—C13	124.46 (10)
N1—C6—H6	124.9	O3—C16—C13	111.99 (9)
C7—C6—H6	124.9	O3—C17—H17A	109.5
C6—C7—C8	106.17 (9)	O3—C17—H17B	109.5
C6—C7—C10	124.52 (10)	H17A—C17—H17B	109.5
C8—C7—C10	129.22 (9)	O3—C17—H17C	109.5
C9—C8—C5	118.56 (10)	H17A—C17—H17C	109.5
C9—C8—C7	134.66 (10)	H17B—C17—H17C	109.5
			
C1—O1—C2—C9	−1.96 (15)	C5—C8—C9—C2	2.07 (14)
C1—O1—C2—C3	177.66 (9)	C7—C8—C9—C2	−178.95 (11)
O1—C2—C3—C4	179.99 (9)	C6—C7—C10—C15	−159.36 (10)
C9—C2—C3—C4	−0.38 (16)	C8—C7—C10—C15	24.50 (16)
C2—C3—C4—C5	1.45 (16)	C6—C7—C10—C11	21.27 (15)
C6—N1—C5—C4	−179.04 (11)	C8—C7—C10—C11	−154.88 (10)
C6—N1—C5—C8	−0.29 (12)	C15—C10—C11—C12	−1.09 (15)
C3—C4—C5—N1	177.83 (10)	C7—C10—C11—C12	178.32 (9)
C3—C4—C5—C8	−0.75 (16)	C10—C11—C12—C13	−0.27 (15)
C5—N1—C6—C7	−0.45 (13)	C11—C12—C13—C14	0.69 (15)
N1—C6—C7—C8	0.98 (12)	C11—C12—C13—C16	179.85 (9)
N1—C6—C7—C10	−175.91 (9)	C12—C13—C14—C15	0.28 (15)
N1—C5—C8—C9	−179.88 (9)	C16—C13—C14—C15	−178.85 (9)
C4—C5—C8—C9	−1.02 (15)	C13—C14—C15—C10	−1.69 (15)
N1—C5—C8—C7	0.87 (11)	C11—C10—C15—C14	2.06 (15)
C4—C5—C8—C7	179.74 (10)	C7—C10—C15—C14	−177.33 (9)
C6—C7—C8—C9	179.80 (11)	C17—O3—C16—O2	−1.53 (14)
C10—C7—C8—C9	−3.51 (19)	C17—O3—C16—C13	177.50 (8)
C6—C7—C8—C5	−1.13 (11)	C12—C13—C16—O2	5.35 (15)
C10—C7—C8—C5	175.56 (10)	C14—C13—C16—O2	−175.51 (10)
O1—C2—C9—C8	178.17 (9)	C12—C13—C16—O3	−173.67 (8)
C3—C2—C9—C8	−1.43 (15)	C14—C13—C16—O3	5.47 (13)

### Hydrogen-bond geometry (Å, °)

**Table:**

Cg2 is the centroid ofthe C2–C5/C8/C9 ring.

**Table:**

*D*—H···*A*	*D*—H	H···*A*	*D*···*A*	*D*—H···*A*
N1—H1···Cg2^i^	0.90 (1)	2.54 (2)	3.295 (2)	142.(1)
C6—H6···O1^ii^	0.95	2.43	3.369 (2)	172
C17—H17B···O2^iii^	0.98	2.60	3.484 (2)	150

Symmetry codes: (i) −*x*+1, *y*−1/2, −*z*+3/2; (ii) *x*, −*y*+3/2, *z*+1/2; (iii) *x*, *y*+1, *z*.
